# Structures and Functions of Viral 5′ Non-Coding Genomic RNA Domain-I in Group-B Enterovirus Infections

**DOI:** 10.3390/v12090919

**Published:** 2020-08-21

**Authors:** Marie Glenet, Laetitia Heng, Domitille Callon, Anne-Laure Lebreil, Paul-Antoine Gretteau, Yohan Nguyen, Fatma Berri, Laurent Andreoletti

**Affiliations:** 1 EA-4684 CardioVir, Faculty of Medicine, University of Reims Champagne-Ardenne (URCA), 51097 Reims, France; marie.glenet@gmail.com (M.G.); laetitia.heng@gmail.com (L.H.); domitille.callon@gmail.com (D.C.); anne-laure.lebreil@univ-reims.fr (A.-L.L.); gretteaupa@hotmail.fr (P.-A.G.); ynguyen@chu-reims.fr (Y.N.); fatma.berri@univ-reims.fr (F.B.); 2 Laboratoire de Biopathologie, Centre Hospitalier Universitaire Reims, 51097 Reims, France; 3 Service de Médecine Interne, Immunologie clinique et maladies infectieuses, Centre Hospitalier Universitaire Reims, 51097 Reims, France; 4 Laboratoire de Virologie Médicale et Moléculaire, Centre Hospitalier Universitaire Reims, 51097 Reims, France

**Keywords:** group-B enterovirus, RNA domain-I, viral ribonucleoprotein complexes, enterovirus replication, 5′ terminally deleted viral forms, antiviral innate immune response, type I interferon

## Abstract

Group-B enteroviruses (EV-B) are ubiquitous naked single-stranded positive RNA viral pathogens that are responsible for common acute or persistent human infections. Their genome is composed in the 5′ end by a non-coding region, which is crucial for the initiation of the viral replication and translation processes. RNA domain-I secondary structures can interact with viral or cellular proteins to form viral ribonucleoprotein (RNP) complexes regulating viral genomic replication, whereas RNA domains-II to -VII (internal ribosome entry site, IRES) are known to interact with cellular ribosomal subunits to initiate the viral translation process. Natural 5′ terminally deleted viral forms lacking some genomic RNA domain-I secondary structures have been described in EV-B induced murine or human infections. Recent in vitro studies have evidenced that the loss of some viral RNP complexes in the RNA domain-I can modulate the viral replication and infectivity levels in EV-B infections. Moreover, the disruption of secondary structures of RNA domain-I could impair viral RNA sensing by RIG-I (Retinoic acid inducible gene I) or MDA5 (melanoma differentiation-associated protein 5) receptors, a way to overcome antiviral innate immune response. Overall, natural 5′ terminally deleted viral genomes resulting in the loss of various structures in the RNA domain-I could be major key players of host–cell interactions driving the development of acute or persistent EV-B infections.

## 1. Introduction

Group-B enteroviruses (EV-B) are common human pathogens responsible for a wide range of pathologies such as myocarditis, pancreatitis, hepatitis, or meningitis resulting from acute infections in children and young adults [1,2]. Some part of acute EV-B infections can evolve toward persistent infections responsible for chronic pathologies such as dilated cardiomyopathy (DCM), type 1 diabetes, and inflammatory myopathy [3,4,5,6,7].

**Figure 1:**
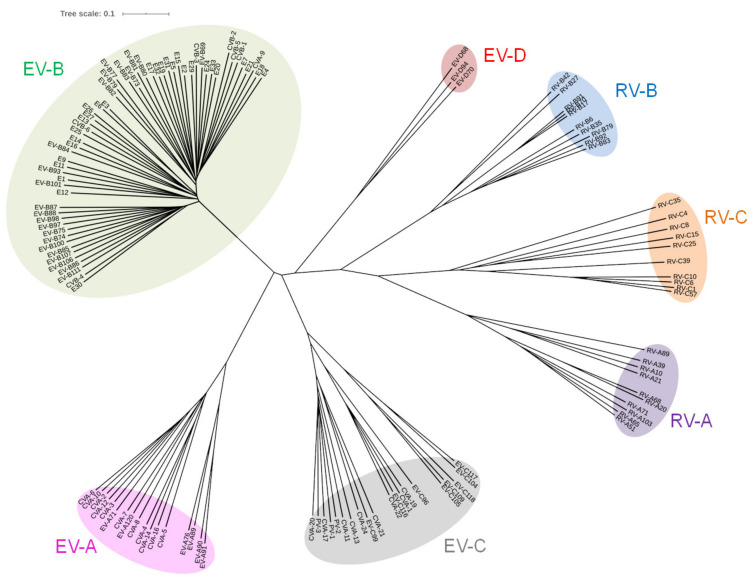
Evolutionary relationship of human enterovirus species. Molecular phylogenetic analysis of human enteroviruses was inferred using the Neighbor-Joining method (Letunic and Bork, 2019). Phylogenetic tree was constructed using complete sequence of enteroviruses aligned by muscle method. The optimal tree with the sum of branch length = 18.13314604 is shown. The tree is drawn to scale, with branch lengths in the same units as those of the evolutionary distances used to infer the phylogenetic tree. The evolutionary distances were computed using the Kimura 2-parameter method and are in the units of the number of base substitutions per site. This analysis involved 128 nucleotide sequences. All ambiguous positions were removed for each sequence pair (pairwise deletion option). There was a total of 8522 positions in the final dataset. Evolutionary analyses were conducted in MEGA X (Kumar et al., 2018). EV-A: Enterovirus A; EV-B: Enterovirus B; EV-C: Enterovirus C; EV-D: Enterovirus D; RV-A: Rhinovirus A; RV-B: Rhinovirus B; RV-C: Rhinovirus C.

**Figure 2:**
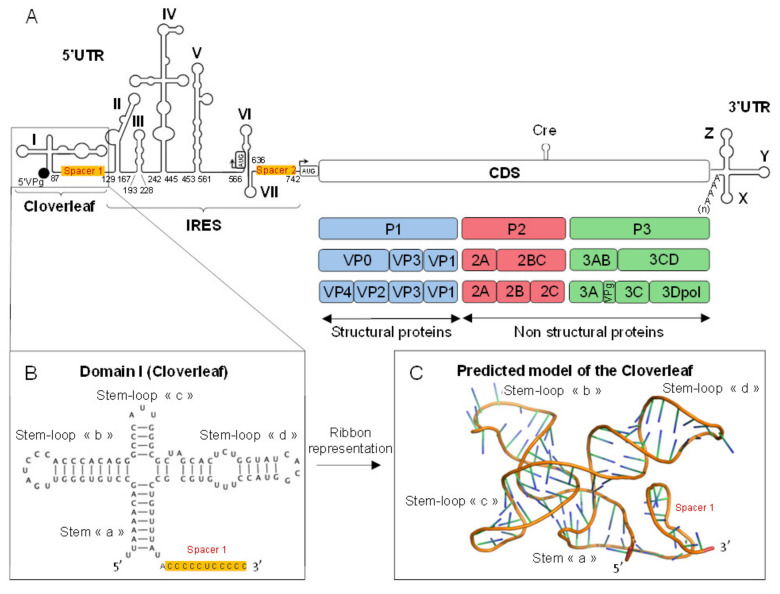
Group-B enterovirus genome organization. (**A**) Enterovirus-B viral genome is composed of two open reading frames (ORFs) which one is a polyprotein coding for capsidic (viral proteins 1 to 4) and non-structural viral proteins such as proteases 2A, 3C, and the 3D polymerase. This long ORF is flanked by two untranslated regions (UTRs): the 5′ UTR is composed of a Cloverleaf-like secondary structure (CL, stem-loop I) responsible for the viral genomic replication separated by a spacer sequence comprising two C-rich clusters from an Internal Ribosome Entry Site element type 1 (IRES, stem-loops II to VII) which has a role in the viral translation. Nucleotide numbers are indicated below the structures. (**B**,**C**) Schematic representation of the predicted 5′ end Cloverleaf secondary structures (CL, stem-loop I) of the enteroviral positive strand RNA. The CL is composed of a stem “a” (nucleotides 2–9 with nucleotides 80–87), stem-loop “b” (nucleotides 10–34), stem-loop “c” (nucleotides 35–45), and a stem-loop “d” (nucleotides 46–79).

EV-B belong to the *Picornaviridae* family [8] and the *Enterovirus* genus is composed of 15 species, classified according to their biological properties and phylogenetic analyses, seven of which are human viruses: EV-A to -D species and three rhinoviruses species (RV-A to -C) (Figure 1) [9,10,11]. EV-B are small naked single-stranded positive RNA viruses of approximately 7400 nucleotides (nt) [12]. Enteroviral genome is functionally divided in four parts (Figure 2A) with a 5′ non-coding region (5′ NCR), two open reading frames (one monocistronic coding region), and a 3′ non-coding region (3′ NCR) [13]. The 5′ NCR is crucial for the initiation of the replication and translation of the viral genome. This enteroviral 5′ NCR is about 750 nt in length and composed of two secondary structure complexes: the first one is involved in the replication of both viral positive- and negative-strands and is organized in one stem “a” and three stem-loops “b”, “c”, and “d” resulting in a 5′ end Cloverleaf-like structure commonly named viral RNA domain-I (Figure 2B). The second one is composed of six stem-loops (domains-II to -VII) forming an internal ribosome entry site (IRES) involved in cap-independent polyprotein translation.

The viral replication process is well-conserved among EV species [14]. Following the viral capsid attachment to various cellular co-receptors or receptors defining the cell tropism, the positive-strand RNA is released from the viral particle into endocytoplasmic vesicles [14]. The capsid-free viral positive single-stranded RNA is directly translated in a cap-independent mRNA structure according to the cellular ribosomes’ recruitment to its large type 1 IRES (domains-II to -VII) into two open reading frames (ORFs). The viral polyprotein is then cis-cleaved by its autocatalytic 2A (2A^pro^) and 3C proteases (3C^pro^) into structural viral proteins 1–4 (VP1–4) and non-structural proteins including the RNA polymerase [15]. The single positive-stranded RNA replicates through a negative-strand intermediate. Generation of the negative-strand RNA is dependent on RNA sequences and secondary structure domain-I interacting with viral and cellular proteins to form viral ribonucleoprotein (RNP) complexes. Enteroviral genomic replication mechanisms result in the efficient synthesis of negative single-strand RNA used as a matrix to then generate positive single-strand RNA.

Enteroviral RNA genomes are known to mutate more easily than DNA viruses, thus leading to mutated and deleted forms of the viral RNA including the 5′-terminally deleted (5′ TD) viral genomes. Natural 5′ TD viral genomes resulting in the loss of different structures ranging from stem “a”, to stem-loop “b” or “c” or part of stem-loop “d” in the 5′ terminal Cloverleaf RNA structure (domain-I) were described in in vitro and in vivo experimental models of EV-B infections. Recently, EV-B populations with 5′ terminal genomic RNA deletions (5′ TD) similar to those described in experimental models were associated with the development of myocarditis or DCM human cases [16,17]. These 5′ TD EV-B populations were characterized as low replicating RNA forms with viral-encoded proteinase activities related with dystrophin cleavage in human cardiac tissues [16]. Through the transfection of synthetic 5′ TD EV-B RNA forms into murine and human cardiac cells, experimental in vitro results evidenced that the loss of some RNP units in viral RNA domain-I could modulate the viral replication and infectivity levels during early phases of EV-B target cell infections [18]. Altogether these results indicate that the loss of some secondary structures of RNA domain-I could significantly impair the viral genomic RNA replication activities resulting in a significant decrease of viral fitness and infectivity levels in target tissues [18].

Reported natural deletions within the 5′ NCR of EV-B forms are known to affect functional secondary-structural elements of the RNA domain-I (Cloverleaf-like structure) [18]. The loss of some secondary structures of RNA domain-I could impair the viral genomic RNA recognition by retinoic acid inducible gene I (RIG-I)-like receptors (RLRs: RIG-I and melanoma differentiation-associated protein 5, MDA5) which are critical cellular sensors for innate immune defense against Picornaviruses [19,20]. To date, the specific contributions of RIG-I or MDA5 to the anti-EV-B innate immune response remain to be investigated in various infected cells. Through transfection of synthetic complete or various 5′ TD EV-B RNA forms into cultured human cardiac cells, it was demonstrated that natural deletions into the 5′ RNA domain-I of EV-B can modify the RNA secondary-structural elements recognized by cytoplasmic RLRs such as RIG-I or MDA5 and modulate type I interferon (IFN) activation pathway induction [17]. Interestingly, similar natural 5′ terminally deletions capable of modulating activation type I IFN response were characterized in patients with acute myocarditis. Overall, these findings evidenced that natural 5′ TD RNA forms in EV-B populations can modulate type I IFN induction, a way to overcome antiviral innate immune response and to promote persistent infection in EV-B human target tissues such as heart and pancreas.

The present article reviews the importance of secondary structural elements of 5′ terminal EV-B RNA domain-I for the formation of viral RNP complexes regulating viral genome replication and also for the activation of type I IFN signaling pathway by RLRs (RIG-I, MAD5) sensors. The present review deciphers functional consequences of the natural loss of some viral 5′ terminal RNA domain-I secondary structures on the viral RNA replication and on the type I IFN pathway activation in EV-B infected human cells. Overall, natural 5′ TD viral genomes resulting in the loss of different structures in the RNA domain-I could be major key players of host–cell interactions driving the development of acute or persistent infections in human target cells.

## 2. Functional Organization of 5′ Terminal EV-B RNA Secondary Structures

EV-B replication activities are dependent on the 5′ NCR of about 750 nt in length divided into two highly conserved and well-known structured functional regions. The first one is a ≈90 nt predicted Cloverleaf-like secondary structure also known as stem-loop-I (SL-I) or domain-I, which is crucial for the initiation of the genomic replication for both viral positive- and negative-strands RNA. Enteroviral RNA domain-I presents a conserved structural pattern among EV-B organized in one stem “a*”* and three stem-loops “b”, “c”, and “d” and commonly named Cloverleaf-like structure or RNA domain-I (Figure 2B,C). The second two-dimensional structured pattern from the 5′ NCR is composed of six stem-loops (SL II–VII) and is an IRES involved in the cap-independent viral polyprotein translation (Figure 2A) [21,22,23,24], the host range [25], the replication efficiency [26,27], the cell tropism, and the virulence [26,28,29]. Two regions of around 40 and 100 nt space, respectively, the Cloverleaf from IRES and then the IRES from the translation initiation codon into the VP4 coding sequence [30] (Figure 2A). Enteroviral genome has a principal ORF encoding a long and single monocistronic autocatalytic polyprotein of ≈250 kDa resulting in viral capsid proteins and non-structural proteins. Moreover, a second upstream ORF (uORF) of the principal has been recently identified and could encode for a protein promoting the viral growth in initial viral replication sites (human epithelial cells) and facilitating viral particle release [13,31]. These two ORFs are flanked by 5′ and 3′ NCRs, also called untranslated regions (UTR). The genomic RNA of picornaviruses is linked to a viral protein genome-linked (VPg) at its 5′ NCR, which acts as a primer during viral RNA synthesis [32,33,34,35]

## 3. Enteroviral 5′ Terminal Domain-I RNA Sequences Involved in Viral Genomic Replication Through the Formation of Viral Ribonucleoprotein Complexes

**Figure 3:**
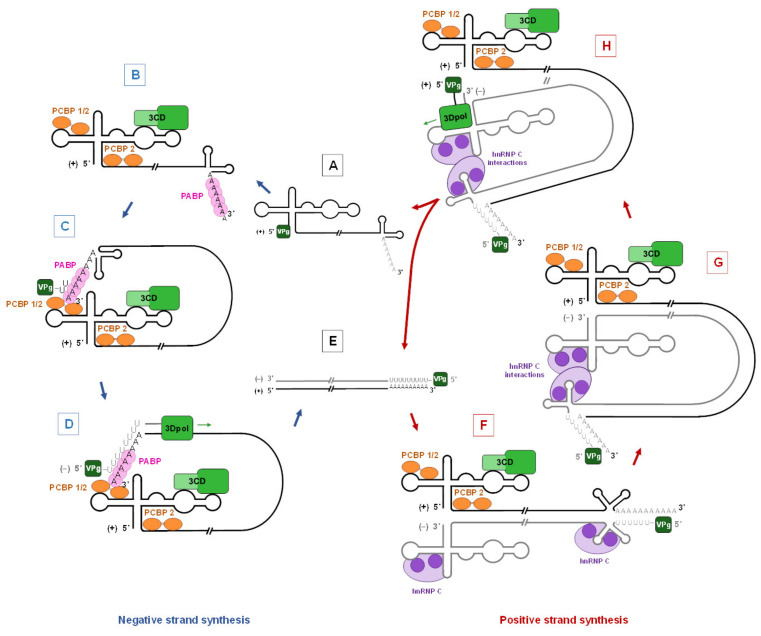
Viral ribonucleoprotein complexes involved in group-B enterovirus genomic replication process. (**A**) The enterovirus genome is single-stranded positive RNA with a 5′ non-coding region (5′ NCR) including a Cloverleaf-like structure (RNA domain-I). The genomic RNA is linked to a viral protein genome-linked (VPg) at its 5′-terminus, which acts as a primer during viral RNA synthesis. (**B**) Host cellular protein Poly(A) binding protein (PABP) interacts with the poly(A) tail in the viral genome 3′ end; Poly(C) Binding Protein (PCBP) 1/2 binds to the stem-loop “b” of the Cloverleaf and PCBP 2 binds to the spacer 1. Viral precursor protein 3CD interacts with stem-loop “d” of the Cloverleaf. (**C**) Through protein–protein interactions between PCBP 1/2 and PABP, the 5′ ends interact to form a ribonucleoprotein complex (RNP). The circularization of the viral RNA initiates the negative-strand synthesis. (**D**) The viral 3D polymerase synthesizes the negative strand. (**E**) The product of the negative-strand synthesis is a double-strand RNA complex called replication form. (**F**) Viral double-strand RNA complex unwinds at both ends, which enables the two Cloverleaf-like structures to interact with cellular and viral proteins. Nuclear protein, heterogeneous nuclear RNP-C (hnRNP-C), can interact with both ends of the negative-strand. Interactions of viral double-strand RNA complex with cellular and viral proteins allow maintaining a single-stranded structure at both ends of the replication form. (**G**) hnRNP-C could promote negative-strand circularization by oligomerization. (**H**) The circularization allows the initiation of the positive-strand synthesis. The 3D polymerase recruitment allows the start of positive-strand synthesis using VPg as primer. This process results in the formation of neo-synthetized positive- strands RNA and viral double-strand RNA complexes.

Enteroviral RNA secondary structure domain-I interacts with viral and cellular proteins to form viral RNP complexes mediating viral genome replication mechanisms in infected cells (Figure 3). The Cloverleaf structure, first described in polioviruses, is a cis-acting RNA replication element required for initiation of negative- and positive-strand synthesis [36,37,38,39,40,41].

Following the attachment and the decapsidation phases of enteroviral cycle, the viral RNA of positive-polarity is directly translated into the cytoplasm and allows the synthesis of proteins necessary for its own genomic replication and the production of new virions. After translation of viral proteins, the first step of viral genome replication consists in the transcription of the positive-polarity RNA by 3D polymerase (3D^pol^) into an RNA of negative polarity (also called antigenomic or replication intermediate), which will be used as a template for the synthesis of new viral RNA of positive-polarity that can be translated in turn (Figure 3).

Negative-strand RNA synthesis involves the formation of a functional RNP complex at the 5′ end of the viral genomic RNA. The host cellular poly(C) binding proteins 1 and 2 (PCBP1/2) and poly(A) binding proteins (PABP) are major key factors of the viral minus-strands RNA synthesis [36]. Briefly to construct this RNP, cellular cytoplasmic PCBP1/2 interact with the stem-loop “b” of the Cloverleaf structure whereas the viral 3CD proteinase (3CD^pro^) interacts with the stem-loop “d”, while PABP binds to the poly(A) located at the end of the 3′ UTR [42,43] (Figure 3B and Table 1). Moreover, PCBP2 interacts with the spacer 1 at the end of the Cloverleaf-like structure (Figure 3B). The binding of PABP to PCBP1/2 by protein–protein interactions allows viral RNA folding which results in the circularization of the genomic RNA promoting interactions between the 5′ and 3′ ends for picornaviruses negative-strand RNA synthesis [44] (Figure 3C). Moreover, the 3CD^pro^ interacts with the *cre,* which is a RNA stem-loop element in the 2C coding region [45,46]. This regulating RNA structure enhances 3D^pol^ uridylylation of the viral protein VPg [38,47], which then acts as a primer in negative- and positive-strand RNA synthesis initiation [45,48]. Following the construction of this viral RNP complex, 3D^pol^ exercises its RNA-dependent RNA polymerase activity using the uridylylated VPg as a primer of negative-strand RNA synthesis (Figure 3D). The negative-strand synthesis results in a double-strand RNA complex called replication form (Figure 3E). The protein components of the RNP and their roles fully depend on their binding and interaction sites (Figure 3B, Table 1). The preservation of the Cloverleaf, a cis-acting replication element at the 5′ end of the positive genomic RNA strand through strong selection pressure might be necessary to maintain the primary, secondary, or tertiary structures of this genomic region. Moreover, the existence of a conserved RNA structure has been hypothesized allowing for the replication complex to specifically recognize enteroviral genome RNA among cellular mRNAs [49].

The initiation of positive-strand RNA synthesis involves the formation RNP complexes on the replicative form, which unwinds at both ends allowing to the Cloverleaf to interact with cellular and viral proteins. Cellular factors PCBP1/2 and viral 3CD^pro^ interact, respectively, on the stem-loop “b” and the stem-loop “d” of the Cloverleaf-like structure in the 5′ NCR of the positive-strand viral RNA [41,74], while nuclear protein Heterogeneous nuclear ribonucleoprotein C (hnRNP-C) binds to both ends of the negative-strand [69,96] (Figure 3F and Table 1). Interactions at both ends of the viral double-strand RNA complex with proteins prevent base-pairing with complementary sequence and preserve a single-stranded structure at both ends of the replication form [41]. hnRNP-C oligomerization could, by drawing both ends into proximity, induce negative-strand circularization (Figure 3G). This process results in a stabilized tertiary structure of RNP complex, which allows the initiation of the positive-strand synthesis by the 3D polymerase with uridylylated VPg as primer (Figure 3H). By these successive molecular phases, neo-synthetized positive-strands RNA and viral double-strand RNA complexes are synthesized (Figure 3A,E). During positive-strand RNA synthesis, a large excess of viral positive-strand RNA is simultaneously synthesized from only one negative-strand (60–70-fold increased levels).

Interestingly, hnRNP-C interactions with the 3′ end of EV negative-strand RNA promote the viral proteins recruitment to an initiation complex for positive-strand RNA synthesis, increasing the amplification of genomic RNA [69,96]. Interactions between hnRNP-C and the 3′ end of the EV negative-strand RNA could stabilize the partially duplexed RNA in the replication form by binding of uridylylated VPg, which is an essential step for prime elongation by the 3D^pol^ on the intermediate negative-strand RNA template for positive-strand RNA synthesis [69]. Consequently, lack of full-length ends of negative-strand RNA would induce a decrease in interactions between the 5′ and 3′ ends of negative-strand RNA and ribonucleoproteins resulting in a decreased efficiency of the polymerase to synthesize viral positive-strand RNA [96]. Loss of such negative-stranded viral RNA binding sites might contribute to the significant reduction of positive-strand RNA synthesis and aberrant low (+)/(−) viral RNA ratios as described in murine or human tissues chronically infected by persistent viral populations [16,97]. Moreover, low expression levels of replication complex cellular factors such as hnRNP-C could decrease viral genome replication activity levels of full-length ends of negative-strand viral RNA in various human target tissues and cells [18,98]. Overall, hnRNP-C or other unknown host proteins of RNP complexes formed with the 3′ ends of negative-strand could provide an early replicative advantage of full-length viral RNA forms in specific cells and drive the development of enteroviral human infections [17,18,96]. Identification of new cell host proteins of viral RNP complexes could help in designing new therapeutic strategies either targeting or inhibiting host protein activities involving EV-B RNA replication in human infections. 

## 4. Role of Viral Ribonucleoprotein Complexes in the Regulation of Viral Translation and Genomic Replication Processes

Recruitment by RNA domain-I of various host cell proteins in RNP regulates viral translation and genomic replication processes [99]. The Cloverleaf binds PCBP1/2, which facilitates its interaction with the viral 3CD^pro^ [50] and which, in the case of PCBP2, is required for both translations and viral RNA synthesis initiation in infected cells [51]. Moreover, cleavage of PCBP2 by the 3CD^pro^ contributes to viral translation inhibition [100]. The 5′ NCR of EV-B interacts with hnRNP-K protein which is important for viral replication [64]. Moreover, the Cloverleaf-like structure composed of the initial 1–100 nt region in the 5′ UTR and the IRES of coxsackievirus B3 (CV-B3) are known to interact with Polypyrimidine tract-binding protein (PTB)-associated splicing factor (PSF) [101]. Additionally, the IRES structure of picornaviruses interacts with La, Sam68 (68 kDa Src-associated protein in mitosis), PTB, and SRp20 (Serine/arginine-rich splicing factor) host proteins improving translation [53,102,103,104,105]. On the other hand, interactions of ARE/poly(U)-binding/degradation factor 1 (AUF1), Gemin5, and far-upstream element-binding protein 2 (FBP-2) with the 5′ NCR of picornaviruses negatively regulate viral translation [66,106,107,108]. The functional significance of these various interactions for the progression of enteroviral infection in human cells remains to be investigated. 

## 5. Natural 5′ Terminal Deletions in EV-B RNA Domain-I During Experimental or Human Infections

EV-B are well known for their high genomic plasticity mainly related to the lack of proofreading activity of the 3D^pol^ resulting in approximately one mutation per 10^3^–10^5^ copied nt [109] in every new genome generated during replication phase [110]. The acquisition of mutations (insertions, substitutions, or deletions) allows the generation of a set of viral sub-populations characterized in quasi-species [111,112]. Among these quasi-species, major EV-B populations were shown to contain 5′ terminal deletions ranging up to 49 nt, increasing in deletion size inside RNA domain-I over viral culture passage or time post-infection in experimental models [113]. Various characterized deletions at the end of the 5′ NCR of EV-B RNA affect the secondary structure, resulting in the loss of stem “a”, stem-loop “b”, “c”, and part of stem-loop “d” of the Cloverleaf structure. The related produced virions could still perform the critical functions of cell infection with low levels of genome replication and viral translation activities [75,114]. Interestingly, these 5′ TD viruses showed long-term viral persistence in vitro and in mice heart or pancreas tissues [113,114].

Similar 5′ NCR deleted viral RNA forms have been reported in EV-B induced cardiac human pathologies as myocarditis and DCM [113,114,115,116]. The first clinical characterization of nucleotide deletions in EV-B 5′ NCR was reported in 2008 in endomyocardial biopsy tissues of a CV-B2 induced fulminant viral myocarditis, demonstrating the presence of viral RNA forms deleted 22–36 nt in the 5′ NCR [115]. Recently using deep sequencing strategies, EV-B RNA forms harboring 5′ terminal deletions up to 50 nt were found either alone or present in majority (≈95%) with minor full-length viral forms (≈5%) in heart tissues of a cohort of adult patients with EV-B induced DCM. While 5′ TD viral forms are known to present major defects in viral RNA replication, in vitro experimental results showed that co-transfection of 95% 5′ TD forms with 5% of full-length genome forms into human cardiomyocytes produced higher viral RNA replication activities than transfection with full-length forms alone. In an interesting way, long-term viral replication after this transfection of the mixture led to a lower viral genome synthesis than with those produced by the full-length form alone [16], suggesting a helper function to the minor (full-length) population preserving a functional negative-strand replication but a defect of positive-strand synthesis without any impairment in viral structural and non-structural protein expression [16].

Described 5′ NCR deleted EV-B RNA forms would be defective viral genomes (DVGs) as previously defined with other RNA viruses such as influenza viruses, Sindbis virus, or Sendai virus [117,118]. DVGs are naturally produced viral RNA genomes [119] that displayed three described forms: deleted, copyback, and snapback [120,121]. These truncated forms are naturally produced in the infected host cell and the presence of a helper viral full-length genome (parental genome) is required to perform efficient viral replication activities. These truncated viral RNA forms will compete and decrease parental form replication levels [122]. Interestingly, these DVG forms can overcome the adaptive immune system that activation might clear parental full-length viral forms [123]. Overall, DVG forms could promote the development of high viral genetic diversity levels resulting in the generation of rapidly evolving major populations of viral quasi-species by comparison with the minor parental full-length viral RNA forms [124]. The dynamics of emergence and the impact of DVG or truncated forms onto the enteroviral replication mechanisms and the activation of the innate immune system during early phases of viral infection remains to be investigated.

## 6. Impact of Natural 5′ Terminal Deletions in Viral RNA Domain-I on EV-B Replication Activities

**Figure 4:**
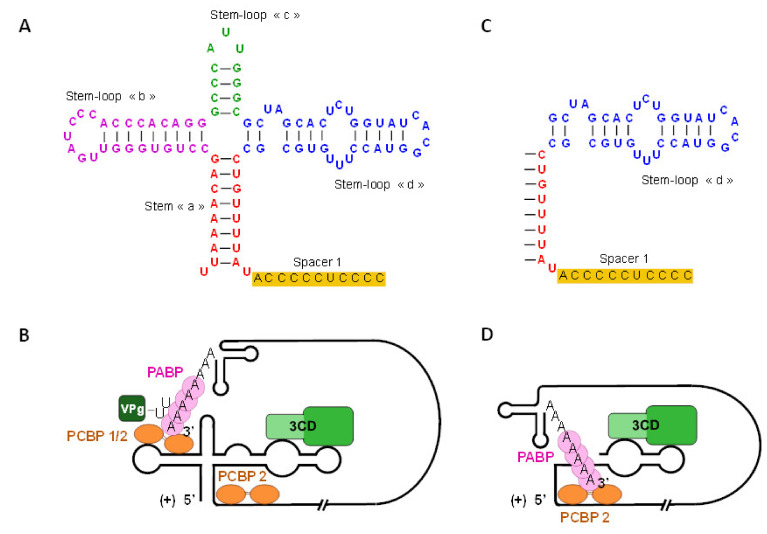
Natural 5′ terminal deletions in RNA domain-I disrupt viral ribonucleoprotein complexes involved in group-B enterovirus replication activities. (**A**) Schematic representation of the complete predicted 5′ end Cloverleaf secondary structures (CL, stem-loop I) of the enteroviral positive-strand RNA. The CL is composed of a stem “a” (nucleotides 2–9 with nucleotides 80–87), stem-loop “b” (nucleotides 10–34), stem-loop “c” (nucleotides 35–45), and a stem-loop “d” (nucleotides 46–79). (**B**) Host cellular protein PABP interacts with the poly(A) tail in the viral genome 3′ end, PCBP 1/2 bind to the stem-loop “b” of the Cloverleaf and in addition, PCBP 2 binds to the spacer 1 at the end of the Cloverleaf. Viral precursor protein 3CD interacts with stem-loop “d” of the Cloverleaf. (**C**) Schematic representation of the 5′ terminally deleted Enterovirus RNA with 50 nucleotides deletions in Cloverleaf secondary structures. (**D**) 5′ terminal deletions in RNA domain-I disrupts the formation of viral ribonucleoprotein complexes: PABP interacts with the 3′ end poly(A) tail, PCBP 2 binds to the spacer 1 at the end of the Cloverleaf, and the precursor 3CD interacts with stem-loop “d” of the Cloverleaf. This loss of interactions with viral protease and cellular factor in stem-loop “b” alters EV-B replication activities.

Partial deletions of the 5′ RNA domain-I sequences significantly disrupt the binding sites of proteins involved in the EV-B RNA synthesis possibly providing a molecular explanation for the low levels of 5′ TD RNA replication in target cells (Figure 4). Investigations by RNA mobility shift assays indicate that the binding of host protein PCBP2 and viral protein 3CD^pro^ to the 5′ end of various deleted forms of positive-strand RNA was qualitatively conserved for the 5′ TD viruses independently of the size of the deletion. However, the stability or protein binding affinity of the RNP complexes decreased with the increase in size of the deletion [18] (Figure 4D). These recent in vitro findings obtained in cultured murine and human cardiac cells provide a possible explanation for the low levels of RNA replication of deleted strains as consequences of the deletions on the formation of RNP complexes. In addition, these data indicate that these low replicative 5′ TD RNA forms may be EV-B persistent populations similar to those observed in clinical studies on heart tissues of patients with unexplained DCM [18].

Interestingly, experimental genetic restoration of the partially deleted Cloverleaf-like secondary structure of described 5′ TD viruses allowed the re-establishment of the viral replication and cellular pathogenesis evidenced through the induction of cytopathic effect on cultured cells [115]. Disrupting PCBP2 binding to either stem-loop “b” or the C-rich sequence inhibits enteroviral RNA replication identifying PCBP2 cell host protein as one of the main cofactors during viral RNA replication [51,54,55] (Figure 4B,D). Within replication complexes, the presence of at least one PCBP binding site in the 5′ NCR (i.e., stem–loop “b” or C-rich sequence) appeared to be sufficient to support efficient negative-strand synthesis [83,85].

Despite different losses of secondary structures and binding sites of viral or host cell proteins in different known viral RNP complexes, natural 5′ TD forms up to 50 nt show a conservation of the stem-loop “d” structure allowing PCBP2 binding to C-rich sequence spacer 1, which is sufficient to support positive and negative strand synthesis at low levels [75] (Figure 4C,D). An unstable RNP complex requiring higher PCBP2 and 3CD^pro^ concentrations sustains 5′ TD RNA low-level replication. These 5′ terminally deletions from 30 to 49 nt induce in silico the tripartite complex formation (PCBP2/3CD^pro^/Domain-I) at 50–60% efficiency compared to that observed with the full-length viral RNA [18]. Overall, partial deletions of this RNA domain up to the stem-loop “c” but preserving the stem-loop “d” resulted in significant decrease levels of viral infectivity by the production encapsidated negative-stranded RNA [18]. These deletions disrupt viral and host protein binding modulating the dynamics of enteroviral infection and potentially driving the development of acute or persistent infections in human target cells. In recent clinical investigations, these naturally generated low replicating deleted viral RNA forms were identified as major populations in human acute myocarditis and chronic DCM cases [16]. In cultured human cardiomyocytes, the deleted viral RNA forms can produce viral proteinases as 2A^pro^ whose levels impact on cell host protein synthesis machinery by cleavage of major host cell proteins as eukaryotic translation initiation factor 4G (eIF4G) [16,18,125,126]. Overall, natural 5′ TD viral genomes resulting in the loss of different structures in the RNA domain-I could be major key players of host–cell interactions in EV-B human infections [17]. 

## 7. Impact of Natural 5′ Terminal Deletions in EV-B RNA Domain-I on Type I Interferon Signaling Pathway Activation

The innate immune system plays an essential role in pathogen recognition and initiation of the immune response through the recognition of pathogen-associated molecular profiles (Pathogen Associated Molecular Pattern, PAMPs) by its Pattern Recognition Receptors (PRRs). The viral nucleic acids, RNAs, or DNAs are recognized as PAMPs. PRRs are essential elements in the detection and induction of the innate response to pathogens including viruses. Recently various PRRs sensors of immunity were identified as Toll-Like Receptors (TLRs), RLRs including RIG-I, MDA5 and LGP2 (Laboratory of Genetics and Physiology 2), and NOD-Like Receptors (NLRs). RLRs have a conserved domain called CTD (C-Terminal Domain), which is responsible for attachment specificity to viral RNAs, whether they are double-stranded RNAs, single-stranded RNAs, or RNAs with a tri-phosphate group at the 5′ end. This C-terminal domain is a repressor domain (RD) which inactivates the caspase recruitment domains (CARD domains) and whose negative regulatory function has only been observed for the RIG-I and LGP2 receptors. The recognition specificity of virus families makes it possible to characterize RLRs; RIG-I is activated by numerous viruses such as Paramyxoviruses, Orthomyxoviruses, Rhabdoviruses, Flaviviruses, and Hepatitis C virus, while MDA5 mainly recognizes viruses of the *Picornaviridae* family and more specifically group-B Enteroviruses, including group-B Coxsackieviruses [127,128]. In order to limit the activation of the host innate immune response by genome RNA sequences, enteroviruses developed various strategies of direct interference on the sensing of cytosolic immune receptors as RLRs during the early phase of viral replication.

**Figure 5:**
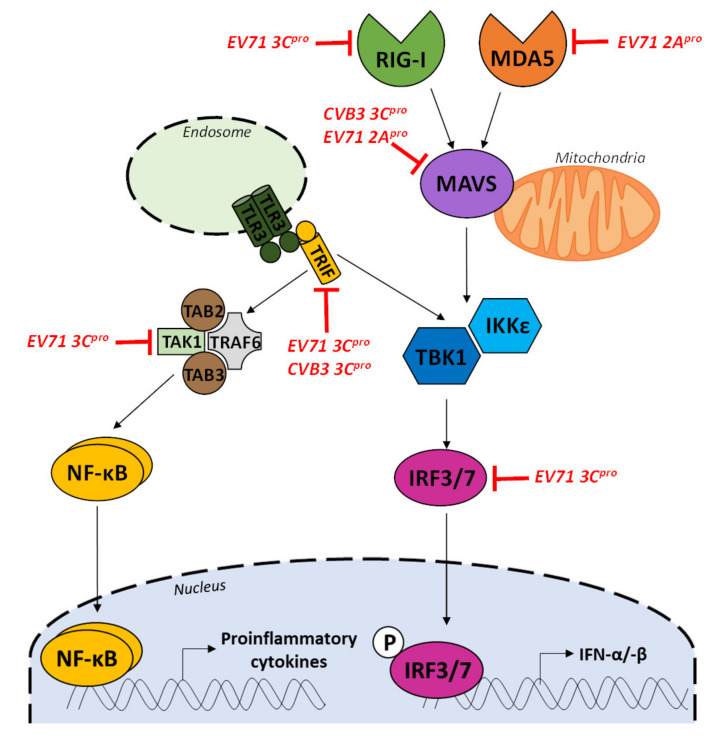
Group-B enterovirus proteinase activities impair type 1 signaling pathway activation in infected cells. Enterovirus proteinase 3C (3C^pro^) and 2A^pro^ are mainly involved in downregulation of type I IFN, pro-inflammatory cytokines at different stages. The interacting cellular signaling molecules with different viral proteins are indicated at each level. (MDA5: melanoma-differentiation-associated protein 5, RIG-I: retinoic acid-inducible gene 1, MAVS: mitochondrial antiviral-signaling protein, TBK1: TANK-binding kinase 1, IRF3/7: Interferon Regulatory Factor 3/7, TRIF: TIR-domain-containing adapter-inducing interferon-β, TLR: toll-like receptors, TRAF6: TNF receptor-associated factor 6, TAK1: transforming growth factor-β activated kinase 1, TAB2/3: TGF-β Activated Kinase 2/3, IFNs: interferons).

A reported strategy developed by EV-B to overcome antiviral immune response is to cleave or partially disrupt RLRs MDA5 receptor during EV-B infection by viral proteases activities, therefore impairing the innate immune sensing of viral RNA double-strand forms [127,129,130,131,132] (Figure 5). For poliovirus, CV-B3, and EV-71, the 2A^pro^ activity results in the disruption of MDA5 structures, even though some authors discuss that such MDA5 or mitochondrial antiviral signaling protein (MAVS) cleavage may take place in a proteasome-dependent or activated caspase-3 pathway [129,133,134]. To transfer the signal downstream in the type I IFN signaling pathway, ligand-bound MDA5 (CARD) links to the adaptor molecule MAVS at the mitochondrial membrane (Figure 5). In a similar way, the 3C^pro^ of EV-B can cleave various intermediate protein factors of the type I IFN pathway [135] including RIG-I receptor (Figure 5) [133,136]. Indeed, 3C^pro^ activity can mediate the cleavage of key molecules such as transforming growth factor-β activated kinase 1 (TAK1)/TGF-β Activated Kinase 1 (TAB1)/TAB2/TAB3 complex which are known to activate the Nuclear factor kappa B pathway inducing a pro-inflammatory cytokine expression [137] (Figure 5). Moreover, EV-71 3C^pro^ activity was described as capable to cleave Interferon Regulatory Factor 7 (IRF-7) inhibiting its ability to transactivate IFN-β expression by its phosphorylated form during early phase of viral infection [133] (Figure 5).

**Figure 6:**
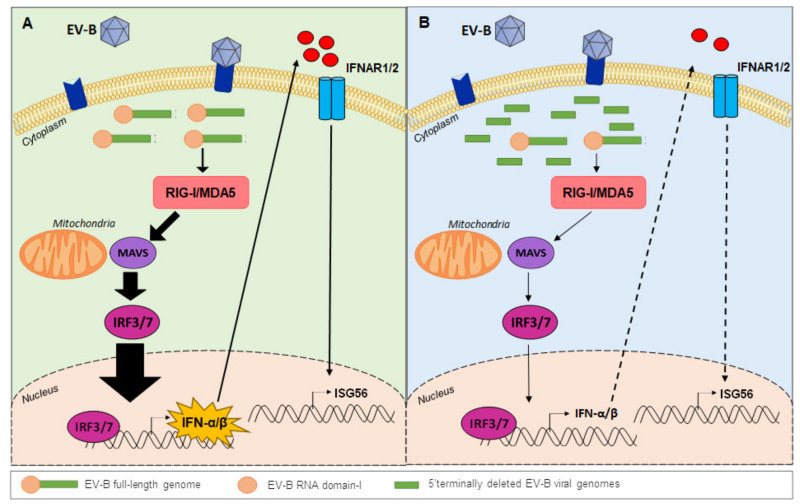
Natural 5′ terminally deleted group-B enterovirus RNA forms can modulate type I interferon signaling pathway activation. (**A**) Full-length viral RNA is recognized by cytoplasmic sensors RIG-I or MDA5 in EV-B infections. Signaling through the adaptor protein MAVS leads to IRF3 activation and translocation to the nucleus. These molecules stimulate high level of IFN-α/β and ISG56 production for the development of effective antiviral responses to EV-B infections. (**B**) According to recent reports, deleted viral genomes were associated with parental complete virus in early phase group-B enterovirus infection. Host cell proteins recruited in viral RNP complexes or potentially acting as a restriction factors could provide an evolutionary advantage to the 5′ NCR truncated viral RNA forms. Deletion in RNA domain-I (5′ terminally deleted viral genomes) could impair the viral genomic RNA recognition by RLRs (RIG-I or MDA5) immune sensors during the early phase of antiviral innate immune response resulting in low level of IFN-α/β and ISG56 production. IFN: interferon; RIG-I: retinoic acid-inducible gene-I; MDA5: melanoma differentiation-associated protein 5; MAVS: mitochondria antiviral-signaling protein; IRF: Interferon Regulatory Factor 3; ISG56: interferon stimulated gene 56; 5′ NCR: 5′ non-coding region.

Natural truncated forms of up to 50 nt in the 5′ NCR of the EV-B in experimental or clinical infections [16,116] would allow the maintenance of the viral genome in the infected host cell by a modulated activation of the innate immune system [17] (Figure 6). Recently, these 5′ TD EV-B RNA forms showed their ability, alone, to produce in silico, in vitro, and in vivo viral proteins such as the viral 2A proteinase resulting in 2A^pro^-specific eIF4G cleavage [16]. This suggests that natural 5′ TD forms helped by a full-length RNA virus are capable of replicating their genome and translating their proteins at low levels. These low levels of viral proteinases can lead to the potential cleavage and extinction of viral genome recognition by RLRs. Low replication of 5′ TD EV-B RNA forms associated with low synthesis and activity of 2A^pro^ and 3C^pro^ inducing cleavage of the MDA5 or RIG-I receptors as well as MAVS intermediate remain to be fully elucidated in experimental in vitro and in vivo models.

EV-B RNA domain-I secondary structures in the 5′ UTR may be critical in the host recognition of the viral genome by RLRs, which is crucial for triggering an host innate immune response [20,133] (Figure 6A). Previous studies demonstrated the importance of some nucleotide variations in the 5′ NCR, showing that deletions or point mutations affect the secondary structures of EV-B RNA domain-I [99]. The loss of one or several secondary structures of RNA domain-I could impair the viral genomic RNA recognition by RLRs (RIG-I or MDA5) immune sensors during the early phase of antiviral innate immune response or could contribute to the virus ability to escape the innate immune response [99] (Figure 6B). This hypothesis is supported by a recent report demonstrating that two major 5′ TD viral RNA population groups were identified in peripheral blood or heart tissue samples of EV-B acute myocarditis cases and that their respective proportions appeared to be positively or negatively correlated with type I IFN levels [17]. To confirm immunomodulatory effects of these 5′ TD RNA forms on the type I IFN pathway induction, synthetic CV-B3/28 RNAs harboring various 5′ terminal full-length or deleted genomic sequences were produced by the authors and transfected into human primary cardiomyocytes cultures [17]. This experimental approach demonstrates that EV-B genomic RNA domain-I possesses essential immunomodulatory secondary-structural elements responsible for IFN-β pathway induction in human cardiomyocytes. Natural 5′ terminally RNA deletions disrupting secondary structures of RNA domain-I could result in a differential qualitative binding of EV-B 5′ TD RNA sequences to the RLRs (RIG-I or MDA5) acting as major innate immune sensors during EV-B acute infections (Figure 6). Modulation of RLRs activation by 5′ terminal RNA structures of EV-B 5′ TD viruses could impair type I IFN pathway activation, thus regulating IFN-β and interferon-stimulated gene (ISG) transcription and translation levels in target cells, a way to overcome antiviral innate immune response [17] (Figure 6).

## 8. Involvement of Natural 5′ Terminally Deleted EV-B RNA Forms in Persistent Infections

RNA viruses including picornaviruses have been shown to develop persistent infections in human target tissues [120,138,139,140]. Among human picornaviruses, polioviruses and group-B coxsackieviruses have been shown to establish persistent infections in target tissues resulting in chronic pathologies such as type 1 diabetes and DCM [120]. Cellular and molecular mechanisms underlying establishment and maintenance of persistent EV-B infections remain unknown. In vivo, specifically in chronic myocarditis or DCM, EV-B persistent infection has been defined by several associated viral criteria including: a low viral RNA load (<3 log_10_ genome copies per microgram of total nucleic acids extracted), an absence of detected infectious particle by classical culture assays, a plus- to minus-strand viral RNA ratio <5 and the detection of a viral protein synthesis activity [141]. Emergence and maintenance of persistent EV-B forms during acute infection depend on early host–virus interactions. 

Emergence of 5′ TD viral populations has been proposed as a molecular mechanism for enterovirus persistence in vivo [116,140]. The association of minor full-length RNA and major 5′ TD viral populations has been found in mouse models and in human persistent cardiac infections [16,114,116]. Full-length EV-B RNA population could help 5′ TD RNA forms replication by genomic recombination processes, sustaining synthesis of negative-strand RNA but not positive-strand [18,142]. Such a scenario would lead to low viral loads, accumulation of double-strand and minus RNA complexes, and undetectable infectious particles which are hallmarks of viral persistence [140,143].

Maintenance of the viral genome is a key step to establish viral RNA persistence in target cells [19]. 5′ TD RNA forms associated with long-term viral persistence described above are known for low-levels of replication activity and progeny [16]. These truncated viral populations displayed 5′ NCR deletions in RNA domain-I disrupting secondary RNA structures, which could impair viral RNA sensing by RLRs and result in a modulation of type I IFN expression in target cells [17]. Given that 5′ TD viruses are low replicating forms, the generation or maintenance of such variants could be an efficient process for EV-B to develop a chronic persistent infection in human target cells long after the acute infection phase [16,18,144,145,146,147]. The combination of low-level replication and translation activities of naturally produced 5′ TD viruses, associated with the capacity of these RNA forms to modulate type-I IFN response activation pathway could explain long-lasting detection of EV-B genome in some human target tissues such as heart and pancreas.

## 9. Conclusions and Future Directions

The 5′ NCR of EV-B is involved in key steps of the viral life cycle. The recruitment by RNA domain-I of host cell and viral proteins in RNP complexes is essential for viral translation and genomic replication processes. Natural deletions within the 5′ NCR of EV-B forms affect functional secondary-structural elements of the RNA domain-I. The loss of these secondary structures of RNA Cloverleaf reduces the binding of viral and cellular proteins implicated in EV-B genome replication mechanisms, therefore modulating the viral fitness and infectivity of viral populations. Early emergence of 5′ TD viral populations and their maintenance in infected cells have been recently proposed as a potential molecular mechanism driving the development of EV-B human acute and persistent infections [16,17]. Further research programs will aim to identify unknown host cell proteins recruited in viral RNP complexes implicated in 5′ TD EV-B replication process or potentially acting as restriction factors providing an evolutionary advantage to the 5′ NCR truncated viral RNA forms. A genome wide CRISPR–Cas9 screen in near haploid human HAP1 cells approach could uncover differences between host proteins factors crucial for infection and replication of EV-B full-length or 5′ TD forms in human target cells [148]. Identification of new cell host proteins implicated in viral RNP complexes or unknown host cell restriction factors could be a promising new therapeutic strategy targeting or inhibiting host protein activities involved in either the replication or selection of viral populations with 5′ terminal deletions in viral RNA domain-I.

In addition, to disturb viral genome RNA replication processes, 5’NCR deletions could impair the viral RNA recognition by RLRs, inducing the modulation of type I IFN expression, specifically by regulating IFN-β and ISG transcription and translation levels in target cells [17]. Other unexplored mechanisms developed by EV-B could also overcome antiviral innate immune response. Indeed, post-transcriptional RNA modifications commonly called epitranscriptome, are observed at specific residues of cellular RNA and play a key role in their maturation and their functionality and can determine the sensing of viral RNA sequences by RLRs [149,150,151]. Among the numerous chemical modifications described, the methylation of nucleotides in various nucleotide positions is the most abundant in eukaryotic cells. To date, very few studies have so far been devoted to modifications of viral RNA and despite the identification of certain modifications of RNA present in viruses, little information is known about their roles in infection and spread of viral pathogens. Several modifications of viral RNA appear today fundamental for the regulation of arboviruses (ZIKA and Dengue) and other virus replication and spread [152,153]. The 2′O-Methylation of the viral mRNA cap by West Nile virus evades innate immune response by modulating type I IFN pathway activation [149,154]. However, such post-transcriptional modifications in EV-B RNA and their impact on the activation of type I IFN pathway and more specifically on the viral RNA sensing by RLRs are not yet investigated. Using new detection approaches based on genome-wide high-throughput sequencing, further scientific projects have to map the EV-B RNA post-transcriptional modifications and to investigate their impact on the regulation of innate immune response in the early phase of host cell infection.

Finally, published reports and ongoing scientific investigations highlight the importance of secondary structures of RNA domain-I and related functional RNP complexes in the EV-B infectious cycle and related host cell immune response. Overall, natural 5′ TD viral genomes resulting in the loss of different structures in the RNA domain-I could be major key players of host–cell interactions driving the development of acute or persistent EV-B infections in human target cells.

## Figures and Tables

**Table 1:** Cellular and viral factors involved in the formation of ribonucleoprotein complexes in group-B enterovirus genomic replication or translation process.

Cellular/Viral Factors		Binding Site in RNA Structure	Interaction Partner	Role in EV-B Replication Activities	References
PCBP 1	Poly(C) Binding Protein 1	SL-I “b” (Cloverleaf) and reduced affinity to SL-IV (IRES)	Viral 3C proteases (3C^pro^) and 3CD^pro^	Initiation of the viral translation and RNA synthesis	[50,51,52]
PCBP 2	Poly(C) Binding Protein 2	SL-I “b”, C-rich spacer) and SL-IV (IRES)	SRp20 (Serine/arginine-rich splicing factor), PCBP2 dimerization, viral 2A^pro^, 3C^pro^, and 3CD^pro^	Initiation of the viral translation and RNA synthesis. Switch from viral translation to viral genome replication	[50,52,53,54,55,56,57,58,59,60]
EF-1α	Eukaryotic elongation factor 1α	SL-I	Viral 3CD^pro^	Cofactor candidate of the enteroviral genome replication	[61]
La	Lupus autoantigen	Reduced affinity to SL-I, IRES, and 3′ UTR	La dimerization	5′ UTR-La/La-3′ UTR complex may form a replication loop and enhance viral replication initiation. Cofactor of the pre-translation initiation protein-RNA complex	[62,63]
hnRNP K	Heterogeneous nuclear ribonucleoprotein K	SL-I to II and SL-IV (IRES) and biotinylated EV71 5′ UTR	K Homology (KH) 2 domain, the proline-rich domain, and one neighboring KH domain (KH1 or KH3)	Inhibition of viral RNA synthesis	[64,65]
hnRNP A1 & A2	Heterogeneous nuclear ribonucleoprotein A1 and A2	SL-II to II and SL-VI (IRES)	-	Activation of IRES activity/regulation of alternative splicing, and it antagonizes the activity of serine-arginine rich (SR) family proteins	[66,67,68]
hnRNP C	Heterogeneous nuclear ribonucleoprotein C	Positive-strand SL-V, negative-strand 5′ and SL-I “a” of 3′ ends	Viral P2 and P3 and 3D^pol^, 3CD^pro^	Enhance viral RNA synthesis. Stabilize and promote efficient positive-strand RNA synthesis	[69,70,71]
PABP	Poly(A) binding protein	SL-I “b” (Cloverleaf) and Poly(A) tail	PCBP2, 3C^pro^, and 3CD^pro^	Enhance IRES-mediated translation and RNA synthesis. Genome circularization for the initiation of negative strand RNA synthesis	[39,44,72]
3AB	Protein 3AB, precursor of viral proteins 3A and VPg (viral protein genome-linked)	SL-I “b” (Cloverleaf) complexed with 3CD^pro^	Viral protease 3C and 3CD in absence of RNA	Enhance IRES-mediated translation and RNA synthesis. Destabilize the secondary structures of RNA, enhance its hybridization in viral replication and 3D^pol^ stimulation	[73]
3CD^pro^	Proteinase 3CD, precursor of viral 3C^pro^ and 3D^pol^	SL-I “d” (Cloverleaf) and 3′ UTR and Cis acting replication element (cre)	3AB, PABP1/2, EF-1α, hnRNP C, PABP	Shut off of the cellular transcription, protease activity, circularization of the viral genome and complex formation for RNA replication	[36,74,75,76,77,78,79,80,81,82]
3C^pro^	Proteinase 3C	SL-I “b” or “d” (Cloverleaf)	PABP, PCBP2	Cleavage of cellular proteins like translation initiation factor eIF4G and PABP leading to shut off of host translation + cleavage of hnRNP A1 leading to reduce inhibitor effect of hnRNP A1 on apoptosis + initiations of the viral translation and RNA synthesis	[36,56,80,82,83,84,85,86,87,88]
FBP1	Far-upstream element-binding protein 1	Only linker region (nt 637 to 745) of the EV71 5′ UTR	FUSE upstream of the c-*myc* gene and KH3 and KH4 motifs	Positive regulatory factor for IRES activity and enhance viral protein synthesis	[65,89]
FBP2	Far-upstream element-binding protein 2	SL I, II region, the SL II, III region, and SL V, VI and linker regions of EV71 5′ UTR	FUSE upstream of the c-*myc* gene and KH2 and KH4 motifs	Negative regulatory factor for IRES activity and inhibition of viral protein synthesis	[65,89]
2A^pro^	Proteinase 2A	-	Cleave 3CD to produce 3C′ and 3D′ + VP1 and P2	Cleavage of cellular proteins like translation initiation factor eIF4G and PABP leading to shutoff of host translation	[90,91,92,93]
2C & 2BC	2C^ATP*ase*^ and precursor 2BC	SL I “b” (Cloverleaf)	-	Only negative-strand synthesis initiation	[36,94]
3B	VPg (Viral protein genome-linked)	5′ ends	3D^pol^	Primer in both positive- and negative-strand RNA synthesis	[95]
3D^pol^	Viral 3D RNA-dependent RNA polymerase	3′ UTR	3B, 3AB, and 3D^pol^	Elongation activity	[61]

EV-B: group-B enterovirus, SL: stem-loop, UTR: untranslated region, IRES: Internal Ribosome Entry Site, FUSE: far-upstream element.
